# Towards a comprehensive barcode library for arctic life - Ephemeroptera, Plecoptera, and Trichoptera of Churchill, Manitoba, Canada

**DOI:** 10.1186/1742-9994-6-30

**Published:** 2009-12-10

**Authors:** Xin Zhou, Sarah J Adamowicz, Luke M Jacobus, R Edward DeWalt, Paul DN Hebert

**Affiliations:** 1 Canadian Centre for DNA Barcoding, Biodiversity Institute of Ontario, University of Guelph, 50 Stone Road East, Guelph, Ontario, N1G 2W1, Canada; 2 Department of Biology, Indiana University, Bloomington, IN 47405, USA; 3 Illinois Natural History Survey, 1816 S Oak St, Champaign IL 61820, USA

## Abstract

**Background:**

This study reports progress in assembling a DNA barcode reference library for Ephemeroptera, Plecoptera, and Trichoptera ("EPTs") from a Canadian subarctic site, which is the focus of a comprehensive biodiversity inventory using DNA barcoding. These three groups of aquatic insects exhibit a moderate level of species diversity, making them ideal for testing the feasibility of DNA barcoding for routine biotic surveys. We explore the correlation between the morphological species delineations, DNA barcode-based haplotype clusters delimited by a sequence threshold (2%), and a threshold-free approach to biodiversity quantification--phylogenetic diversity.

**Results:**

A DNA barcode reference library is built for 112 EPT species for the focal region, consisting of 2277 COI sequences. Close correspondence was found between EPT morphospecies and haplotype clusters as designated using a standard threshold value. Similarly, the shapes of taxon accumulation curves based upon haplotype clusters were very similar to those generated using phylogenetic diversity accumulation curves, but were much more computationally efficient.

**Conclusion:**

The results of this study will facilitate other lines of research on northern EPTs and also bode well for rapidly conducting initial biodiversity assessments in unknown EPT faunas.

## Background

Despite 250 years of taxonomic effort and the description of about 1.7 million species, it is believed that most species remain undescribed [1,2]. Perhaps even more importantly, barriers to the identification of the known species are substantial. Most groups are studied by a few specialists, and even specialists regularly encounter specimens that cannot be reliably identified because diagnostic traits only reside in a particular sex or life stage. Because of such complexities, there is no region on the planet where a comprehensive registry of biodiversity is currently possible. The present study aims to break this barrier by bringing a molecular tool and taxonomic expertise to bear on a single geographic region to construct a reference library of DNA barcodes [a short standardized gene fragment used for species identification, see 3]--both to connect life stages and sexes and to create an identification system based on DNA sequences that is easily transmissible to anyone with access to sequencing technology.

This study represents the first in a series that has the goal of assembling a DNA barcode library for all eukaryote species at Churchill, Manitoba. This site was selected for analysis because its fauna has seen substantial taxonomic and ecological studies, reflecting its location as the most easily accessible site in the Canadian subarctic. Furthermore, its northerly position should aid the assembly of a comprehensive library because species richness [4] and genetic diversity [5] are lower here than at more southerly sites. An additional factor promoting the use of this site lies in the fact that Churchill is home to one of the major research facilities in the Canadian subarctic, both enabling our work and ensuring that a barcode library will gain broad usage in the support of other research. Despite the factors facilitating analysis, the assembly of a comprehensive barcode library for all eukaryote species at this site will require a substantial effort, as the local biota may include as many as 10,000 species.

The present investigation begins the assembly of a barcode library at Churchill by examining 3 of the 15 insect orders which occur there: Ephemeroptera (mayflies), Plecoptera (stoneflies), and Trichoptera (caddisflies) ("EPTs"). Although the earliest studies on these groups began at Churchill more than 60 years ago [6], the species count is modest. Just 13 mayfly, 16 stonefly, and 18 caddisfly species have been reported from the Churchill area. The present study has the primary goal of assembling a comprehensive barcode library for EPT species at this site. We additionally test how the molecular delineation of COI (mitochondrial cytochrome *c *oxidase subunit I) barcode haplotype clusters compares with the morphological species concept and how any difference may affect the construction of taxon accumulation curves, useful tools for assessing biodiversity and indicating the completeness of biotic surveys [7]. Through this work, we provide a first insight into the feasibility of applying DNA barcoding in biodiversity registration of a region even if taxonomic expertise is unavailable. Updated species checklists, detailed discussions of species boundaries, and faunistic analysis are considered in a companion paper [8].

## Results

### Barcoding analyses and DNA barcode reference library

A total of 1500, 564, and 213 COI sequences were recovered from 1644, 565, and 227 Trichoptera, Ephemeroptera, and Plecoptera specimens, respectively (see Table S1 in Additional file 1). The failure to generate sequences for 9% of Trichoptera and 6% of Plecoptera specimens was not correlated to taxonomic identity, as the failures involved individuals belonging to morphospecies with sequence records in the dataset. Thus, no additional optimization of analytical protocols was undertaken for these two groups. The success rate in generating COI sequences in mayflies rose from 89.4% to 99.8% upon employing the newly designed reverse mini primer (MEPTR1-t1, see Materials and Methods). For example, its use reversed the consistent failure of PCR amplification in one mayfly species, *Paraleptophlebia praepedita *(Eaton), that was encountered using routine barcoding primers (LepF1/LepR1 and LCO1490/HCO2198).

Detailed information about each voucher specimen (taxonomic assignment and identifier, repository, collection details, image, COI sequence, and tracefiles) is available in the Barcode of Life Data System [9] in a series of publicly accessible projects 'Ephemeroptera of Churchill', 'Plecoptera of Churchill', and 'Trichoptera of Churchill 2002/2004/2005/2006/2007'. COI sequences are also published in GenBank under accession numbers GU113533-GU115809.

### Barcode divergence and species diversity

**Figure 1:**
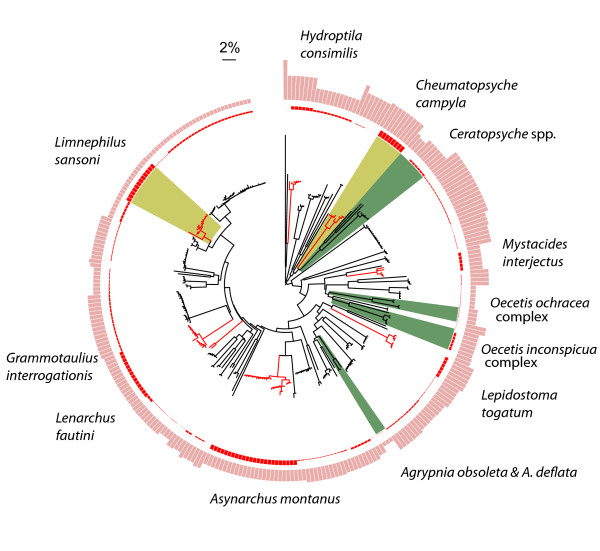
**Circular Neighbour-Joining tree for unique haplotypes of Trichoptera analyzed in this study**. A total of 293 haplotypes represent 1500 COI sequences and 68 morphospecies of Trichoptera. Terminal branches of species with intraspecific divergence greater than 2% are highlighted in red. Species groups with subtle diagnostic characters or females/immatures that are difficult to identify morphologically are highlighted in green blocks. Members of these groups can easily be confused or neglected in routine morphological sorting, but are readily detected via barcoding. Red and pink bars in the outer circles represent the maximum intraspecific divergence and minimum distance to nearest neighbour, respectively, of the corresponding species shown in the circular tree. The heights of the two distance bars are proportional to the distance values (see Table S3 in Additional file 3). Two possible cryptic caddisfly species, *Limnephilus sansoni *and *Cheumatopsyche campyla *(Ross), are highlighted in yellow blocks.

**Figure 2:**
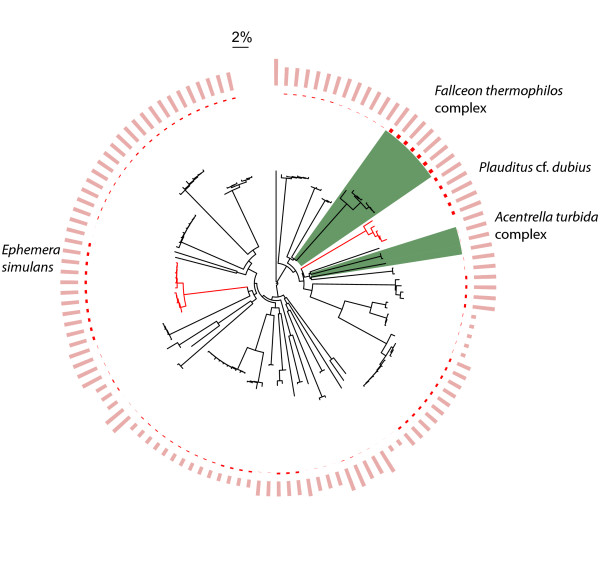
**Circular Neighbour-Joining tree for unique haplotypes of Ephemeroptera analyzed in this study**. A total of 123 haplotypes represent 564 COI sequences and 37 morphospecies of Ephemeroptera. Figure symbols and annotations follow those in Fig. 1.

**Figure 3:**
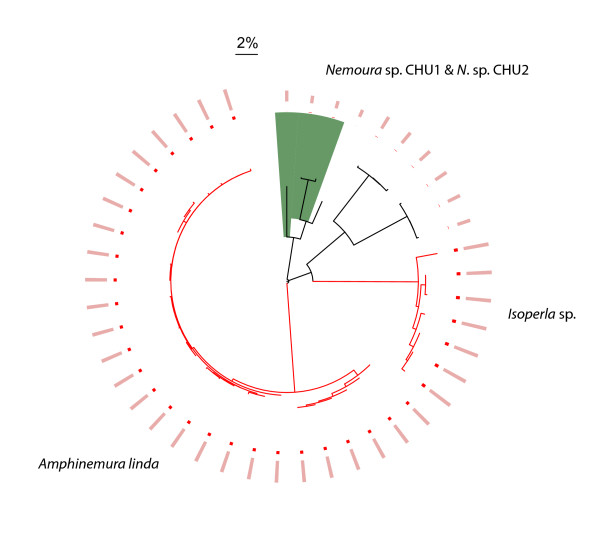
**Circular Neighbour-Joining tree for unique haplotypes of Plecoptera analyzed in this study**. A total of 44 haplotypes represent 213 COI sequences and 7 morphospecies of Plecoptera. Figure symbols and annotations follow those in Fig. 1.

Sixty-eight caddisfly, 37 mayfly, and 7 stonefly morphological species were discovered during this study, the majority of which were new records for the region. We found that members of 7 species groups, representing 16 morphospecies, were easily confused in field sorting due to subtle diagnostic characters or uncertainty in the identification of females or larvae (Figs. 1, 2 and 3, highlighted in green blocks.  See Table S2 in Additional file 2 for distance values). However, these taxa were readily detected by barcodes and their species status was supported by further morphological scrutiny [8].

Deep interspecific divergences at COI were present between most Churchill EPT morphospecies (Figs. 1, 2 and 3, and see Table S3 in Additional file 3). Sharing of barcodes by different species was not observed, and all morphospecies formed monophyletic clusters in the NJ tree. However, several morphospecies possessed relatively high intraspecific COI divergences (those with intraspecific divergences larger than 2% are highlighted in red on terminal branches, Figs. 1, 2 and 3). The search for morphological differences between these deeply divergent lineages was not productive. However, many clusters in question were represented by only a few identifiable individuals (typically males), prohibiting a comprehensive search for diagnostic morphological characters. Although these deeply divergent barcode haplogroups may reflect cryptic species, additional sampling of specimens and/or independent molecular markers is required to draw solid conclusions [10].

In all species except the caddisfly *Limnephilus sansoni *(Banks) (Fig. 1, highlighted in yellow block), no overlap of the maximum intraspecific divergence and the minimum distance to its nearest neighbouring taxon was observed (Figs. 1, 2 and 3, represented in red and pink bars in outer circles, respectively). In 74%, 78%, and 86% of the Trichoptera, Ephemeroptera, and Plecoptera species, respectively, the minimum distance to the nearest neighbour was more than ten-fold greater than the maximum intraspecific divergence, including species with relatively high intraspecific divergence.

### Barcode clusters vs. morphological species

**Figure 4:**
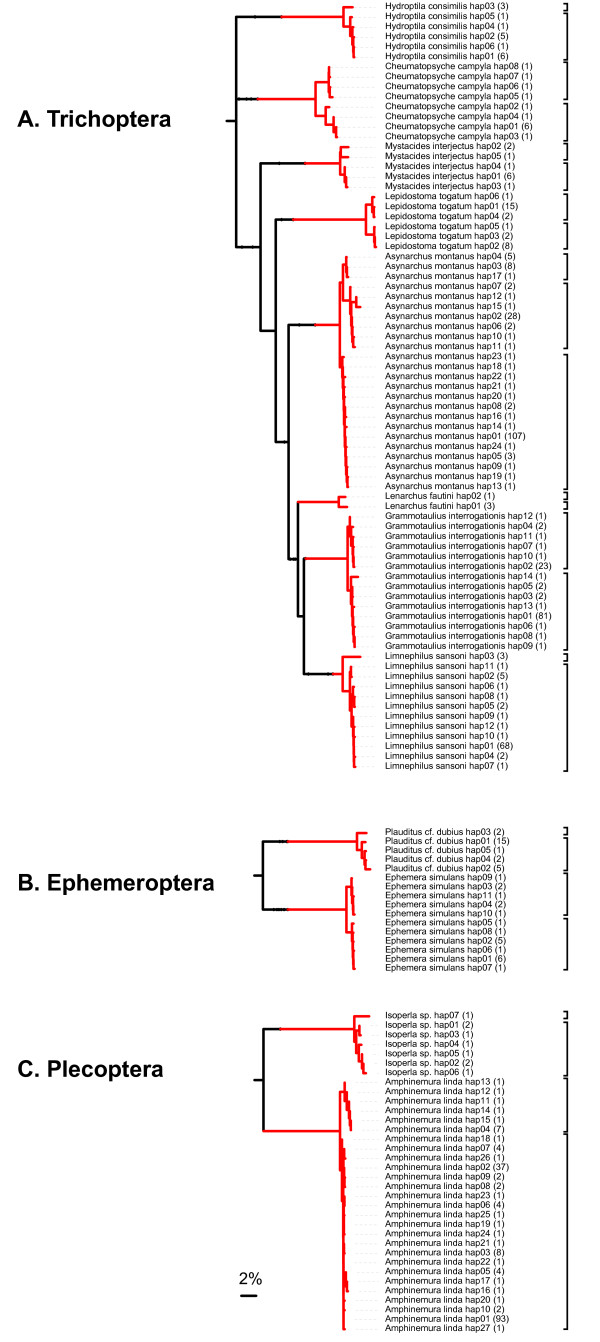
**Barcode haplogroups (2% threshold) compared to morphospecies assignments in species with >2% intraspecific divergence in COI barcodes (in all other cases, the two delineation methods agreed with each other)**. Clades of focal species were detached from the original haplotype NJ trees presented in Figs. 1, 2 and 3 and reassembled to a pruned tree using the Interactive Tree of Life [29]. The number in parentheses next to the haplotype name indicates the number of sequences sharing the haplotype. The square brackets to the right of the tree represent the haplogroup delineated by a 2% threshold.

**Figure 5:**
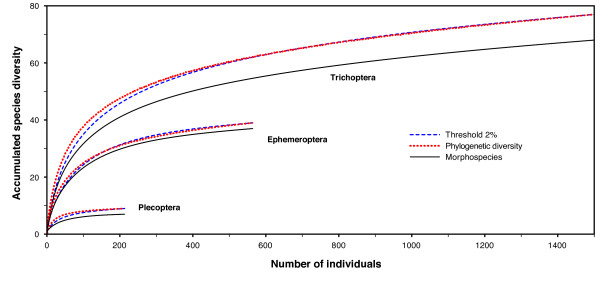
**Randomized taxon accumulation curves based on molecular and morphological taxon delineations and total phylogenetic diversity**. Taxon accumulation curves based on barcode haplogroups and morphospecies were constructed in EstimateS V.8.0 using 50 random replicates and default settings. The phylogenetic diversity (PD) accumulation curve was constructed in R using the packages APE and CAIC (see Methods and Additional file 4). PD values were multiplied by a scaling factor so the end points of the PD and barcode threshold curves would be the same, aiding comparison of the shapes of these curves. As more Churchill EPT individuals are sampled, biodiversity accumulates at a similar rate regardless of how it is quantified, suggesting that all methods applied here could be used in biodiversity assessment. Barcode clusters are more rapidly obtained and efficiently analyzed than morphospecies or total PD.

To test the correlation between barcode clusters and morphospecies (but not to define species, see Materials and Methods), we used an arbitrary 2% threshold to delimit lineages, to be used in the construction of species accumulation curves. This molecular delineation resulted in a similar but not identical conclusion regarding the number of taxonomic entities present in each order (see Table S1 in Additional file 1), with all discrepancies involving the species possessing high intraspecific COI divergences (Fig. 4). For example, there were 68 trichopteran morphospecies but 77 barcode clusters. Species accumulation curves based on barcode clusters and morphospecies were very similar, both in shape and taxon diversity (Fig. 5). These similarities reflect the fact that both methods are measuring nearly the same information in all three taxonomic groups. Thus, for the EPTs of Churchill, barcode clusters delineated with a 2% threshold provide a close estimation to the morphological species concept employed by experienced taxonomists. The rescaled phylogenetic diversity (PD) curves, a threshold-free concept of genetic diversity that is measured by total branch lengths in a tree [[11], see Materials and Methods for details], followed a very similar shape to the species accumulation curves, but rose more rapidly initially and then leveled off, a pattern consistent across the three orders. Finally, the completeness of the biodiversity survey as suggested by the trend of each curve is highly congruent for all three biodiversity quantification methods.

## Discussion

### Assembling the barcode reference library for the Churchill EPTs

The clustering pattern of COI barcodes generated from nearly 2,500 specimens provided a swift overview of Churchill's EPT diversity. DNA barcodes also greatly facilitated the grouping of individuals into putative species, which were subsequently validated through morphological scrutiny by taxonomic experts. Consequently, these COI barcodes have become vouchered reference sequences for the EPT groups at Churchill. Despite the fact that taxonomic revisions may be necessary for several species, e.g., *Oecetis ochracea *(Curtis), *O. inconspicua *(Walker), and *Fallceon thermophilos *(McDunnough) complexes [8], future taxonomic revisions will not compromise the efficiency of species identification using DNA barcodes. Unnamed species and unidentifiable individuals/life stages can also be registered in the barcode library and will be subsequently linked to a specific name when a new species description or identifiable material becomes available.

Although built at one locale, the Churchill EPT barcode reference library is informative for a much broader geographic range of North America. For example, 22 of the 68 Churchill caddisfly species possess Holarctic distributions, and another 21 are broadly distributed in the Nearctic. Many of the latter species occur at southerly sites, reflecting their derivation from lineages that survived the Pleistocene glaciation in southern refugia [12]. Interestingly, the COI sequences for conspecifics from different regions are usually very similar in the EPTs (Zhou, *unpublished*). Of course, the full understanding of the effects of isolation in southern regufia and the role of subsequent postglacial range expansion on COI divergence patterns in widespread Nearctic aquatic insects requires widespread sampling and statistical investigation, which has only been performed in a few taxa [e.g., [13,14]]. Nevertheless, the Churchill barcode reference library remains valuable for identifying EPT species with transcontinental distributions, especially when it is combined with libraries built from samples collected at other geographic localities in North America. These libraries already provide good coverage for the most common species, the ones that are regularly encountered in biodiversity surveys and biological ecosystem assessments. Ongoing targeted surveys in New Brunswick, Ontario, and in the Great Smoky Mountains National Park in Tennessee and North Carolina are contributing to the extension of this continental library.

### A novel approach to registering biodiversity and to revealing new species

Limited access to taxonomic expertise has become a serious impediment to large-scale biodiversity surveys. The present study shows that a comprehensive DNA barcode library built on expertly identified specimens enables fast and accurate species identification by anyone with access to sequencing facilities. Both standard Sanger sequencing for barcoding individual samples and massively parallel pyrosequencing for analyzing bulk environmental samples will undoubtedly become more widespread and less expensive over time, facilitating ecological and monitoring applications of the barcode library. Continued interaction with the taxonomic community during barcode-based biodiversity and monitoring studies, involving submitting specimens with novel sequences for determination or revision, will ensure the growth and maintenance of a high-quality database.

Furthermore, the close correspondence between morphospecies and barcode clusters indicates that biodiversity surveys in new regions need not depend upon immediate access to taxonomic expertise. Instead, preliminary evaluations can be made using barcodes. Obviously, deeper understanding of natural communities--the major goal of biological surveys--requires establishing the linkage between each barcode cluster and its Linnaean name to access the biological information that has been accumulated through past studies. However, barcode sequences alone provide an excellent overview of species diversity, enabling measures of alpha and beta diversities that would otherwise only be available after intensive taxonomic study. Latin names will become available for barcode clusters when new records are compared to existing reference barcode libraries or when no match exists following taxonomic investigation of barcoded specimens. Barcode data can actually speed the process of taxonomic assignment by partitioning a large collection of specimens into a much smaller number of genetic clusters whose taxonomic status can then be assigned. Because of this fact, barcoding will certainly accelerate the construction of taxonomic systems for groups of little-studied life and aid revisionary efforts on lineages that are well known. We see these efforts as complementary.

### To designate species or not

In this study, we employed randomized phylogenetic diversity accumulation curves to test the completeness of our sampling efforts on three insect orders. This approach has several key advantages. Foremost, because it is a threshold-free approach, it makes no assumptions about species boundaries, allowing its application to any group, even those lacking a taxonomic system. As would be expected, PD curves initially rose with a steeper slope than the taxon accumulation curves, whether morphospecies or barcode clusters. This difference reflects the fact that each taxon is treated as equivalent in taxon curves, while more distantly related species contribute more branch length and are, hence, more heavily weighted in the PD analysis. Barring this difference, the different curves had remarkably similar shapes. However, because the calculation of total PD curves is far more computationally intensive, barcode cluster accumulation curves are the more efficient way to assess rapidly the completeness of the biotic surveys.

Prior to drawing a general conclusion about the utility of PD curves in biotic surveys, several additional explorations should be performed. Different taxonomic groups from varied geographical regions should be tested. For example, tropical regions with much greater species diversity should be investigated as they may possess a higher proportion of intermediate branch lengths, due to lower extinction rates. In such settings, the advantages of PD accumulation curves over barcode cluster or threshold-based methods may become apparent. Other taxonomic groups could also display different branching patterns and should be explored. Nevertheless, the pattern of tight clustering within species and much longer branches among species seems to be general across a broad range of animal groups [3,15], including those inhabiting subtropical and tropical environments [16,17].

## Conclusion

This study has generated a DNA barcode reference library for three insect orders--Ephemeroptera, Plecoptera, and Trichoptera at one site in the Canadian subarctic. While we are working towards establishing a comprehensive barcode library for all species in these orders, this library will be immediately useful for biodiversity, ecological, and monitoring studies involving both sexes of adults and all immature stages.

This study has demonstrated that DNA barcoding holds great promise as a tool for rapid biodiversity assessment in unknown faunas. A very close correspondence was observed between morphospecies as determined by taxonomic experts and barcode clusters designated using a standard sequence threshold. Several cases of proposed splitting may reflect cryptic species, which will be explored in a future contribution [8]. Rapid assessment of biodiversity will aid the selection of sites of special conservation value and will help to focus the efforts of taxonomists in revising and characterizing the diversity of life.

## Materials and methods

### Collection site, materials, and identification

Churchill is situated at the confluence of the Churchill River and Hudson Bay [18]. This region is characterized by a long, harsh winter and short flight season for aquatic insects [6]. The area represents an ecotone between northern boreal forest and subarctic tundra habitats.

Mayfly, stonefly, and caddisfly specimens were collected in the Churchill region from 2002 to 2007. Average daily air temperatures at Churchill in June and September are typically lower than 7°C [19], conditions that are unsuitable for flight by most caddisflies [20]. As a result, most collecting activities for caddisflies occurred from late June to late August of 2006 and 2007. Mayfly samples were intensively collected during the last two weeks of July in 2007, while stoneflies were contributed by researchers conducting various bio-surveillance projects in this area from mid-June to mid-August of 2006 and 2007. A wide range of lentic and lotic habitats were sampled, including the Churchill River, tundra ponds, lakes, small streams, and pools on rock bluffs near the margin of Hudson Bay.

Adult samples were collected using UV light traps, aerial nets, Malaise traps, and pit-fall traps. Larval samples were collected using a kicknet and by handpicking. Adult specimens were pinned or preserved in 95% ethanol while all larval samples were kept in 95% ethanol. EPT specimens are deposited in the Biodiversity Institute of Ontario, University of Guelph, at the University of Manitoba, and in the University of Minnesota Insect Collection.

Sequencing of COI barcodes for most EPT samples collected during 2002-2006 was conducted before taxonomic experts became involved so all individuals in the collections, including dominant species, were sequenced. Therefore, DNA barcodes should largely reflect the relative abundances of species in the obtained samples. Specimens were subsequently sorted into groups based on their COI clustering patterns. Morphological identification was carried out independently for each of the barcode cluster series after DNA analysis. Additional EPT specimens collected in 2007 were identified before DNA analysis and were combined with the library.

### DNA analysis and sequence analysis

COI sequences were generated at the Canadian Centre for DNA Barcoding, University of Guelph. Standard barcoding protocols were followed [21]. Typically, a single leg was used for the extraction of genomic DNA using an AcroPrep™ 96 1 ml filter plate (PALL) with 3.0 μm Glass fiber. DNA was eluted in 40 μl of dH_2_O. Full-length COI barcodes (658 bps) were amplified using two primer sets: LepF1 (5'-ATTCAACCAATCATAAAGATATTGG-3')/LepR1 (5'-TAAACTTCTGGATGTCCAAAAAATCA-3') [22] and LCO1490 (5'-GGTCAACAAATCATAAAGATATTGG-3')/HCO2198 (5'-TAAACTTCAGGGTGACCAAAAAATCA-3') [23]. MLepF1 and MLepR1 primers [16] were employed when full-length PCR amplification was not successful. A new reverse mini-primer, tagged with a M13 tail, MEPTR1-t1 (5'-CAGGAAACAGCTATGACGGTGGRTATACIGTTCAICC-3') was paired with LCO1490-t1 to recover the first 325 bps of the 5' terminus of barcode region. This primer set proved to be effective in EPTs, particularly mayflies.

Each PCR reaction had a total volume of 12.5 μl and contained 5% trehalose (D-(+)-Trehalose dehydrate), 1.25 μl of 10× reaction buffer, 2.5 mM of MgCl_2_, 1.25 pmol each of forward and reverse primer, 50 μM of dNTP (Promega), 0.3 U of Platinum *Taq *DNA polymerase (Invitrogen), and 2 μl of genomic DNA. PCR products were visualized on a 2% agarose E-gel^® ^96-well system (Invitrogen). Amplification products were sequenced bi-directionally using BigDye v3.1 and analyzed on an ABI 3730xl DNA Analyzer (Applied Biosystems) as described in deWaard *et al. *[24] and Hajibabaei *et al. *[25].

COI sequences were aligned in MEGA 4.0 [26] using the integrated ClustalX method with default parameters. The amino acid translation was examined to ensure that no gaps or stop codons were present in the alignment. Unique haplotypes for each species were recognized using analytical tools available at the "DNA Barcoding Tools" website http://www.ibarcode.org[27]. These haplotypes were then imported into MEGA for tree construction using the Neighbour-Joining method with pair-wise deletion of missing sites and Kimura-2-Parameter (K2P) distance [28] options. A Newick format tree was exported from MEGA and was annotated using an online tool for phylogenetic tree display--Interactive Tree of Life [29]. Genetic distances were obtained using sequence analytic tools ("Nearest Neighbour Summary") provided in BOLD using K2P distances for all sequences longer than 350 bps.

### Testing barcode cluster delineation and the morphological species concept

The morphological identifications employed in this work are based on current nomenclature for each taxonomic group. All valid species are morphologically distinguishable from others and possess consistent diagnostic character sets, even though barcode sequences may show distinctive groups within such morphological species. To aid the discussion, we refer to the units recognized through morphological study as 'morphospecies' throughout this paper.

To test how patterns of genetic divergence at COI correspond to morphological species concepts, we estimated the Churchill EPT species diversity based on the similarity and clustering pattern in their COI barcodes independent of taxonomic assignments. We employed an arbitrary threshold of 2% sequence divergence to draw boundaries for barcode haplotype clusters. This arbitrary threshold is selected due to the fact that intraspecific divergences observed in a variety of groups rarely exceed this value [see [3,22], and [30]]. Although exceptions have been observed in some taxa [e.g., [31,32]], we emphasize that the species definition used in this work is not based on any genetic threshold, but on concordant evidence from morphology and barcode similarity. We seek only to determine if such a simple delimitation of mitochondrial COI haplogroups for Churchill's EPTs could be informative in evaluating the trend and completeness of general biological sampling, even if taxonomic expertise were not available.

Taxon accumulation curves were constructed to assess the degree of completeness of this survey and to compare the results that would be obtained with and without access to taxonomic expertise. Randomized accumulation curves were built based on morphospecies determined by taxonomists (XZ, LMJ, and RED) and on barcode clusters as delimited using a 2% threshold, using EstimateS V.8.0 [33] with 50 randomization replicates and default settings.

Additionally, the correspondence between these two measures and the total phylogenetic diversity was explored. DNA sequences were formatted for the program R version 2.8.1 [34] and analyzed using the packages APE [35] and CAIC [36]. A Neighbour-Joining tree based upon K2P distances and pair-wise deletion was reconstructed. For each tip number, ranging from 1 up to the total sample size of individuals, tips were randomly sampled 1,000 times. At each replicate, total phylogenetic diversity was calculated and then averaged across randomizations for each tip number. A detailed protocol along with all commands used is provided in Additional file 4. Resulting phylogenetic diversity values were multiplied by a scaling factor to allow their presentation on the same scale as the species accumulation curves, aiding comparison of their shapes.

## Competing interests

The authors declare that they have no competing interests.

## Authors' contributions

XZ and PDNH designed the study; XZ conducted and coordinated field collecting and DNA sequencing; XZ, LMJ, and RED identified specimens; XZ and SJA analyzed the data; and all authors contributed to the writing of the manuscript and approved the final version.

## Supplementary Material

Additional file 1 **Table S1**. EPT specimen voucher information and species delineation using morphospecies identification and 2% threshold. Click here for file

Additional file 2 **Table S2**. Summary of mean and maximum intraspecific divergences and minimum distance to nearest neighbouring taxon. Click here for file

Additional file 3 **Table S3**. Summary of intraspecific divergence and interspecific distances within genus and family. Click here for file

Additional file 4 **Detailed instructions for constructing randomized phylogenetic diversity accumulation curves using the program R**. Click here for file

## References

[B1] Wilson EO The Diversity of Life 1992 Cambridge: Harvard University Press

[B2] Ødegaard F How many species of arthropods? Erwin's estimate revised Biological Journal of the Linnean Society 2000 71 583 597 10.1111/j.1095-8312.2000.tb01279.x

[B3] Hebert PDN Ratnasingham S deWaard JR Barcoding animal life: cytochrome *c *oxidase subunit 1 divergences among closely related species Proceedings of the Royal Society of London Series B-Biological Sciences 2003 270 S96 S99 10.1098/rsbl.2003.0025 PMC1698023 12952648

[B4] Gaston KJ Blackburn TM Pattern and processes in macroecology 2000 Oxford: Blackwell Scientific

[B5] Hewitt G The genetic legacy of the Quaternary ice ages Nature 2000 405 907 913 10.1038/35016000 10879524

[B6] McClure HE Aspection in the biotic communities of the Churchill area, Manitoba Ecological Monographs 1943 13 1 35 10.2307/1943588

[B7] Gotelli NJ Colwell RK Quantifying biodiversity: procedures and pitfalls in the measurement and comparison of species richness Ecology Letters 2001 4 379 391 10.1046/j.1461-0248.2001.00230.x

[B8] Zhou X Jacobus LM DeWalt RE Adamowicz SJ Hebert PDN The Ephemeroptera, Plecoptera, and Trichoptera fauna of Churchill (Manitoba, Canada): insights into biodiversity patterns from DNA barcoding Journal of the North American Benthological Society 2010 in press

[B9] Ratnasingham S Hebert PDN BOLD: The Barcode of Life Data System Molecular Ecology Notes 2007 7 355 364 http://www.barcodinglife.org 10.1111/j.1471-8286.2007.01678.x 18784790 PMC1890991

[B10] Zhou X Kjer KM Morse JC Associating larvae and adults of Chinese Hydropsychidae caddisflies (Insecta: Trichoptera) using DNA sequences Journal of the North American Benthological Society 2007 26 719 742 10.1899/06-089.1

[B11] Faith DP Conservation evaluation and phylogenetic diversity Biological Conservation 1992 61 1 10 10.1016/0006-3207(92)91201-3

[B12] Pielou EC After the Ice Age: The Return of Life to Glaciated North America 1992 Chicago: University of Chicago Press

[B13] Heilveil JS Berlocher SH Berlocher SH Phylogeography of postglacial range expansion in Nigronia serricornis Say (Megaloptera: Corydalidae) Molecular Ecology 2006 15 1627 1641 10.1111/j.1365-294X.2006.02876.x 16629816

[B14] Kauwe JSK Shiozawa DK Evans RP Phylogeographic and nested clade analysis of the stonefly Pteronarcys californica (Plecoptera: Pteronarcyidae) in the western USA Journal of the North American Benthological Society 2004 23 824 838 10.1899/0887-3593(2004)023<0824:PANCAO>2.0.CO;2

[B15] Kerr KCR Stoeckle MY Dove CJ Weigt LA Francis CM Hebert PDN Comprehensive DNA barcode coverage of North American birds Molecular Ecology Notes 2007 7 535 543 10.1111/j.1471-8286.2007.01670.x 18784793 PMC2259444

[B16] Hajibabaei M Janzen DH Burns JM Hallwachs W Hebert PDN DNA barcodes distinguish species of tropical Lepidoptera Proceedings of the National Academy of Sciences of the United States of America 2006 103 968 971 10.1073/pnas.0510466103 16418261 PMC1327734

[B17] Kerr KCR Lijtmaer DA Barreira AS Hebert PDN Tubaro PL Probing evolutionary patterns in Neotropical birds through DNA barcodes PLoS One 2009 4 e4379 10.1371/journal.pone.0004379 19194495 PMC2632745

[B18] Mackay RJ Cushing CE, Cummins KW, Mishall GW River and Stream Ecosystems of Canada River and Stream Ecosystems of the World 2006 Berkeley: University of California Press 33 60

[B19] Environment Canada National Climate Data and Information Archive: Canadian Climate Normals 1971-2000 http://climate.weatheroffice.ec.gc.ca/climate_normals/index_e.html

[B20] Waringer JA Phenology and the influence of meteorological parameters on the catching success of light-trapping for Trichoptera Freshwater Biology 1991 25 307 319 10.1111/j.1365-2427.1991.tb00493.x

[B21] Ivanova NV DeWaard JR Hebert PDN An inexpensive, automation-friendly protocol for recovering high-quality DNA Molecular Ecology Notes 2006 6 998 1002 10.1111/j.1471-8286.2006.01428.x

[B22] Hebert PDN Penton EH Burns JM Janzen DH Hallwachs W Ten species in one: DNA barcoding reveals cryptic species in the neotropical skipper butterfly *Astraptes fulgerator* Proceedings of the National Academy of Sciences of the United States of America 2004 101 14812 14817 10.1073/pnas.0406166101 15465915 PMC522015

[B23] Folmer O Black M Hoeh W Lutz R Vrijenhoek R DNA primers for amplification of mitochondrial cytochrome *c *oxidase subunit I from diverse metazoan invertebrates Molecular Marine Biology and Biotechnology 1994 3 294 299 7881515

[B24] DeWaard JR Ivanova NV Hajibabaei M Hebert PDN Martin CC Assembling DNA barcodes: analytical protocols Environmental Genomics, Methods in Molecular Biology 2008 410 Totowa: Humana Press 275 283 full_text 10.1007/978-1-59745-548-0_15 18642605

[B25] Hajibabaei M DeWaard JR Ivanova NV Ratnasingham S Dooh RT Kirk SL Mackie PM Hebert PDN Critical factors for assembling a high volume of DNA barcodes Philosophical Transactions of the Royal Society B-Biological Sciences 2005 360 1959 1967 10.1098/rstb.2005.1727 PMC1609220 16214753

[B26] Tamura K Dudley J Nei M Kumar S MEGA4: Molecular evolutionary genetics analysis (MEGA) software version 4.0 Molecular Biology and Evolution 2007 24 1596 1599 10.1093/molbev/msm092 17488738

[B27] Singer G Hajibabaei M iBarcode.org: web-based molecular biodiversity analysis BMC Bioinformatics 2009 10 Suppl 6 S14 10.1186/1471-2105-10-S6-S14 19534739 PMC2697637

[B28] Kimura M A simple method for estimating evolutionary rate of base substitutions through comparative studies of nucleotide sequences Journal of Molecular Evolution 1980 16 111 120 10.1007/BF01731581 7463489

[B29] Letunic I Bork P Interactive Tree Of Life (iTOL): an online tool for phylogenetic tree display and annotation Bioinformatics 2007 23 127 128 10.1093/bioinformatics/btl529 17050570

[B30] Ball SL Hebert PDN Burian SK Webb JM Biological identifications of mayflies (Ephemeroptera) using DNA barcodes Journal of the North American Benthological Society 2005 24 508 524

[B31] Alexander LC Delion M Hawthorne DJ Lamp WO Mitochondrial lineages and DNA barcoding of closely related species in the mayfly genus *Ephemerella *(Ephemeroptera: Ephemerellidae) Journal of the North American Benthological Society 2009 28 584 595 10.1899/08-150.1

[B32] Wiemers M Fiedler K Does the DNA barcoding gap exist? - a case study in blue butterflies (Lepidoptera: Lycaenidae) Frontiers in Zoology 2007 8 10.1186/1742-9994-4-8 17343734 PMC1838910

[B33] Colwell RK EstimateS: Statistical estimation of species richness and shared species from samples. Version 8 2006 http://purl.oclc.org/estimates

[B34] R Development Core Team R: A Language and Environment for Statistical Computing 2008 R Foundation for Statistical Computing, Vienna, Austria http://www.R-project.org

[B35] Paradis E Claude J Strimmer K APE: Analyses of Phylogenetics and Evolution in R language Bioinformatics 2004 20 289 290 10.1093/bioinformatics/btg412 14734327

[B36] Orme D Freckleton R Gavin TG Thomas PT CAIC: Comparative Analyses using Independent Contrasts 2008 http://r-forge.r-project.org/projects/caic/18247218

