# Supplementary material for: Towards a comprehensive barcode library for arctic life - Ephemeroptera, Plecoptera, and Trichoptera of Churchill, Manitoba, Canada
Source: Front Zool. 2009 Dec 10;6:30. doi: 10.1186/1742-9994-6-30 (PMC2800108; doi:10.1186/1742-9994-6-30)
Supplement: Additional file 4 — Detailed instructions for constructing randomized phylogenetic diversity accumulation curves using the program R. [file 1742-9994-6-30-S4.DOC]

**Additional File 4: Detailed instructions for constructing randomized phylogenetic diversity accumulation curves using the program R**

For each order separately, aligned sequences were downloaded from their projects in BOLD ([www.barcodinglife.org](http://www.barcodinglife.org/)). They were formatted into a simple text file (.txt extension) that starts with the number of sequences separated by a space from the number of nucleotides. Short sequence titles (truncated sample IDs) were used and were separated by three spaces from the beginning of the sequences according to the following example format:

**562 658**

**seq1 TAC…**

**seq2 AAC...**

…

…

For each of these sequence files, a descriptor file (text file named according to the order analyzed and having the extension .R) was prepared to allow the program R to read in the sequences. These descriptor files contained the following content, as in the example of Ephemeroptera:

**require(ape, quietly = TRUE, save = FALSE)**

**Ephemeroptera <- read.dna("Ephemeroptera.txt", format = "sequential")**

The program R was downloaded (<http://www.r-project.org/>) and installed. The packages ape, MASS, gee, and mvtnorm (all from <http://www.r-project.org/>) and also CAIC (<http://r-forge.r-project.org/projects/caic/>) were downloaded and installed into the "library" folder of R.

The sequence and descriptor files were placed into the “Data” folder within the ape library. One may also specify a path for reading in the data from other locations. Once the R window is opened, copying and pasting in the following commands activated the needed packages:

**library(ape)**

**library(CAIC)**

The following commands instructed R to read in the sequence file for the order Ephemeroptera and to construct a neighbour-joining tree using default (K2P) genetic distances and pair-wise deletion of missing sites. Other types of settings are available (see ape documentation).

**data(Ephemeroptera)**

**tre<-nj(dist.dna(Ephemeroptera,pairwise.deletion=TRUE))**

Next, the resulting tree was formatted into clade matrix format for subsequent analysis.

**clmat <- clade.matrix(tre)**

**clmat**

The next commands provide an example of the phylogenetic diversity resampling procedure. According to these instructions, 5 tips are randomly sampled from the tree. Total phylogenetic diversity (PD), i.e. total branch length, is calculated. This procedure is randomized 100 times and a histogram of the distribution of values is presented. The values are also printed to the screen.

**pd.boot <- pd.bootstrap(clmat, 5, reps=100)**

**hist(pd.boot$pd.distrib)**

**pd.boot$pd.distrib**

In order to construct an accumulation curve, an array was first created to hold the mean PD values for each number of tips from 1 to n-1.

**n<-length(clmat$tip.label)**

**mean.pd<-array(NA,dim=c(n-1))**

A resampling analysis was performed such that each number of tips (from 1 to n-1) was randomly sampled for a certain number of replicates. We used 1000 replicates for each tip number, even though our datasets consisted of up to 1500 individuals. The largest dataset required >24 hours to run on an iMAC with a 2.4 GHz processor and 2 GB of RAM. Depending upon your computer speed, fewer replicates would likely be needed for very large datasets for convenience.

**for (i in 1:(n-1))**

**{pd.boot<-pd.bootstrap(clmat, i, reps=1000)**

**{mean.pd[i]<-mean(pd.boot$pd.distrib, na.rm=TRUE)**

**}**

**}**

The mean PD values for each number of tips was printed to the screen and plotted as an accumulation curve.

**mean.pd**

**plot(mean.pd)**

Finally, we calculated total PD for all tips (no resampling needed), to complete the PD dataset for all tips from 1 to n.

**pd.calc(clmat)**

For the purposes of comparing mean PD accumulation curves with the species accumulation curves, we multiplied the mean PD values by a scaling factor set so that the barcode based haplotype clusters and PD curves would end at the same point. This enabled comparison of their shapes.

Please see the documentation for R and the respective packages for further detail regarding the commands we employed.
